# Genomic Epidemiology of Severe Acute Respiratory Syndrome Coronavirus 2, Colombia

**DOI:** 10.3201/eid2612.202969

**Published:** 2020-12

**Authors:** Katherine Laiton-Donato, Christian Julián Villabona-Arenas, José A. Usme-Ciro, Carlos Franco-Muñoz, Diego A. Álvarez-Díaz, Liz Stephany Villabona-Arenas, Susy Echeverría-Londoño, Zulma M. Cucunubá, Nicolás D. Franco-Sierra, Astrid C. Flórez, Carolina Ferro, Nadim J. Ajami, Diana Marcela Walteros, Franklin Prieto, Carlos Andrés Durán, Martha Lucia Ospina-Martínez, Marcela Mercado-Reyes

**Affiliations:** Instituto Nacional de Salud, Bogotá, Colombia (K. Laiton-Donato, J.A. Usme-Ciro, C. Franco-Muñoz, D.A. Álvarez-Díaz, A.C. Flórez, C. Ferro, D.M. Walteros, F. Prieto, C.A. Durán, M.L. Ospina-Martínez, M. Mercado-Reyes);; Centre for the Mathematical Modelling of Infectious Diseases (CMMID) and London School of Hygiene & Tropical Medicine, London, UK (C.J. Villabona-Arenas);; Universidad Cooperativa de Colombia, Santa Marta, Colombia (J.A. Usme-Ciro);; Universidad Industrial de Santander, Bucaramanga, Colombia (L.S. Villabona-Arenas);; Imperial College-London, London, UK (S. Echeverría-Londoño, Z.M. Cucunubá);; Instituto de Investigación de Recursos Biológicos Alexander von Humboldt, Colombia (N. Franco-Sierra);; Baylor College of Medicine, Houston, Texas, USA (N.J. Ajami)

**Keywords:** respiratory infections, severe acute respiratory syndrome coronavirus 2, SARS-CoV-2, SARS, COVID-19, coronavirus disease, zoonoses, viruses, coronavirus, genome sequencing, phylogenomics, Colombia

## Abstract

Coronavirus disease (COVID-19) in Colombia was first diagnosed in a traveler arriving from Italy on February 26, 2020. However, limited data are available on the origins and number of introductions of COVID-19 into the country. We sequenced the causative agent of COVID-19, severe acute respiratory syndrome coronavirus 2 (SARS-CoV-2), from 43 clinical samples we collected, along with another 79 genome sequences available from Colombia. We investigated the emergence and importation routes for SARS-CoV-2 into Colombia by using epidemiologic, historical air travel, and phylogenetic observations. Our study provides evidence of multiple introductions, mostly from Europe, and documents >12 lineages. Phylogenetic findings validate the lineage diversity, support multiple importation events, and demonstrate the evolutionary relationship of epidemiologically linked transmission chains. Our results reconstruct the early evolutionary history of SARS-CoV-2 in Colombia and highlight the advantages of genome sequencing to complement COVID-19 outbreak investigations.

Coronavirus disease (COVID-19) is a life-threatening respiratory illness caused by severe acute respiratory syndrome coronavirus 2 (SARS-CoV-2), an emerging zoonotic virus first identified in Wuhan, China (*1*). The first confirmed cases of COVID-19 were reported on January 12, 2020, from patients who had respiratory symptoms during December 8, 2019–January 2, 2020 (*2*). Despite early containment and mitigation measures (*3*), the high infectiousness, presymptomatic transmission, and prolonged transmissibility of SARS-CoV-2 (*4*,*5*) combined with other factors, such as globalization, led to the rapid spread of COVID-19 across the world.

Rigorous contact-tracing and physical distancing measures implemented in different countries have been effective in delaying the epidemic during the contention phase (*6*–*9*). However, ensuing lockdowns and travel restrictions to minimize the burden on healthcare systems have led to a decline in wellbeing and an economic downturn and have had profound impacts in low-to-middle income countries (*10*). The contention phase in Colombia started on March 6, 2020, when the Instituto Nacional de Salud (INS; National Institute of Health) confirmed the first case of COVID-19 from a person returning to Colombia from Italy on February 26, 2020 (*11*). On March 23, a total 314 cases had been confirmed, which prompted the closure of all the country borders to contain the outbreak. On March 31, >10% of confirmed cases were among persons with no known exposure to a COVID-19 patient (*12*), presumably due to extensive community transmission. Colombia then implemented the mitigation phase, which included physical distancing as the main strategy to limit virus spread. By June 18, a total of 57,046 confirmed cases and 1,864 deaths had been reported in Colombia (*13*).

The unprecedented global health and societal emergency posed by the COVID-19 pandemic urged data sharing and faster-than-ever outbreak research developments that are reflected in the >37,000 complete SARS-CoV-2 genomes made available through public databases, mainly GISAID (https://www.gisaid.org). SARS-CoV-2 is an RNA virus with an estimated substitution rate of 0.8–1.1 × 10^–3^ substitutions/site/year (S. Duchene et al., unpub data, https://www.biorxiv.org/content/10.1101/2020.05.04.077735v1; M. Worobey et al., unpub. data, https://www.biorxiv.org/content/10.1101/2020.05.21.109322v1), which means it rapidly evolves as it is transmitted. The availability of SARS-CoV-2 genomes enabled us to detect a rapidly generating variation, demonstrating that genomic epidemiology is a powerful approach for characterizing the outbreak (*14*). Genomic epidemiology relies on phylogenetic analysis and has enabled researchers across the world to detect SARS-CoV-2 emergence in humans, reveal the importation and local transmission chains not detected by travel history and traditional contact-tracing strategies, and trace the geographic spread and prevalence of strains bearing specific mutations of epidemiologic relevance (*15**–**17*; S. Dellicour et al, unpub data, https://www.biorxiv.org/content/10.1101/2020.05.05.078758v4; J.R. Fauver et al., unpub data, https://www.medrxiv.org/content/10.1101/2020.03.25.20043828v1). 

## Materials and Methods

### Sample Collection and Preparation

Colombia is made up of 32 departments, which are groups of municipalities, and a capital district. INS received nasopharyngeal swabs samples from patients with clinical signs and symptoms of SARS-CoV-2 from departments across the country as part of the virological surveillance of COVID-19. INS performed quantitative reverse transcription PCR to diagnose suspected COVID-19 cases by using a method recommended and transferred by the Pan American Health Organization and World Health Organization (*18*). Because of scarce resources, we selected a total of 43 samples for genome sequencing that represented >1 of the earliest documented samples in each affected department or samples linked to transmission chains (Appendix 1 Table 1). We performed viral RNA extraction by using the QIAamp Viral RNA Mini Kit (QIAGEN Inc., https://www.qiagen.com) or the MagNA Pure LC nucleic acid extraction system (Roche Diagnostics GmbH, https://lifescience.roche.com).

### Genomic Library Preparation and Sequencing

Library preparation and sequencing were performed following the ARTIC network (https://artic.network) real-time molecular epidemiology for outbreak response protocol and by using both nanopore and next-generation sequencing technologies (*19*). We processed 10 samples by using the MinION sequencer (Oxford Nanopore Technologies, https://nanoporetech.com). We processed the remaining 33 samples by using the Nextera XT DNA library prep kit (Illumina, https://www.illumina.com) and performed sequencing by using the MiSeq Reagent Kit Version 2 and MiSeq sequencer (Illumina).

### Genomic Sequence Assembly

We performed base calling on nanopore reads by using Guppy version 3.2.2 (Oxford Nanopore Technologies) and then demultiplexed and trimmed reads by using Porechop version 0.3.2_pre (*20*). We aligned processed reads against a SARS-CoV-2 reference genome (GenBank reference no. NC_045512.2) by using Burrows-Wheeler Aligner’s Smith-Waterman Alignment (*21*). We performed base calling for single-nucleotide variants with a depth of >200× and then generated polished consensus by using Nanopolish version 0.13.2 (*22*). MiSeq reads were demultiplexed and we used fastp (*23*) to perform quality control using a Q-score threshold of 30. Processed reads were aligned against the SARS-CoV-2 reference genome, we performed bas calling for single nucleotide variants with a depth of >100× and generated consensus genomes by using Burrows-Wheeler Aligner’s Smith-Waterman Alignment version 0.7.17 (*21*) and BBMap (*24*).

### Phylogenetic Analysis of SARS-CoV-2 in Colombia

Sequence data covered the 20 affected departments and the capital district of Colombia. We collected 43 SARS-CoV-2 genome sequences from this study and 79 other sequences from Colombia deposited in GISAID. We combined the 122 sequences from Colombia with 1,461 representative genome sequences from South America–focused subsampling available from NextStrain (https://nextstrain.org) (*25*) as of May 20, 2020 (Appendix 1 Table 2) plus reference MN908947.3 from the GenBank nucleotide database (accesssion no. NC_045512). Across departments, a median of 1.5 sequences (mean 3.9; range 1–45) were available per department. We classified the full genomic dataset into lineages by using Phylogenetic Assignment of Named Global Outbreak LINeages (PANGOLIN) and aligned these with 10 iterative refinements by using MAFFT (*26*–*28*). We removed all alignment positions flagged as problematic for phylogenetic inference, including highly homoplasic positions and 3¢ and 5¢ ends (*29*). We performed maximum-likelihood phylogenetic reconstruction on the curated alignment and a Hasegawa-Kishino-Yano plus gamma distribution 4 substitution model by using IQ-TREE (*30*,*31*). We estimated branch support by using an SH-like approximate likelihood ratio test (SH-aLRT) and considered >0.75 a high SH-aLRT (*32*). We removed 6 sequences from Colombia from further analysis because they had an inconsistent temporal signal in a clock analysis in TreeTime (*33*). We inferred time-scaled trees and rooted these with least-squares criteria and the evolutionary rate of >1.1 × 10^−3^ substitutions/site/year estimated by S. Duchene et al. (unpub data, https://www.biorxiv.org/content/10.1101/2020.05.04.077735v1) by using TreeTime (*33*) and least-squares dating (*34*).

We considered geographic locations of sequence data, aggregated by continent except for Colombia, as discrete states, used these data for migration inference, and modeled transitions as a time reversible process by using TreeTime (*33*). We interpreted the number of state transitions into Colombia as a proxy for the minimum number of introductions.

In sensitivity analysis and to measure the effect of the SARS-CoV-2 uneven genomic representativeness across the world, we implemented 2 downsampling strategy datasets in which, based on location, the sequences were randomly resampled 100 times and the phylogenetic and migration inference was replicated. The downsampling strategies were as follows: retaining several sequences per region, when possible, equal to the number of sequences available for Colombia; or retaining 50 sequences per region and the total number of sequences from Colombia, which was the most even sampling per region for the South America–focused subsample.

### Potential Routes of SARS-CoV-2 Importation into Colombia

We inferred the relative proportion of expected SARS-CoV-2 importations per country by considering COVID-19 incidence per number of international air passengers arriving in Colombia and the available flight travel. We obtained the number of international flights and number of passengers arriving during January 1–March 9, 2020 from the Special Administrative Unit of Civil Aeronautics of Colombia (Aerocivil, http://www.aerocivil.gov.co). The air travel data consists of direct flights from 14 countries to 7 main cities. We calculated COVID-19 incidence for each of the 14 countries with direct flights to Colombia by using the number of confirmed cases reported by the World Health Organization as of March 17, 2020, the date when travel restrictions started in Colombia (*35*), and the total population for each country for 2019 reported in the United Nations World Population Prospects 2019 database (*36*), as described in D.D.S. Candido, et al. (*37*) (Appendix 2).

### Ethics Statement

According to the national law 9/1979, decrees 786/1990 and 2323/2006, the Instituto Nacional de Salud is the reference lab and health authority of the national network of laboratories and in cases of public health emergency or those in which scientific research for public health purposes as required, the Instituto Nacional de Salud may use the biological material for research purposes, without informed consent, which includes the anonymous disclosure of results. The information used for this study comes from secondary sources of data that were previously anonymized and do not represent a risk to the community.

## Results

### Epidemiologic Investigation of SARS-CoV-2 Introductions, Contact-Tracing, and Community Transmission

**Figure 1:**
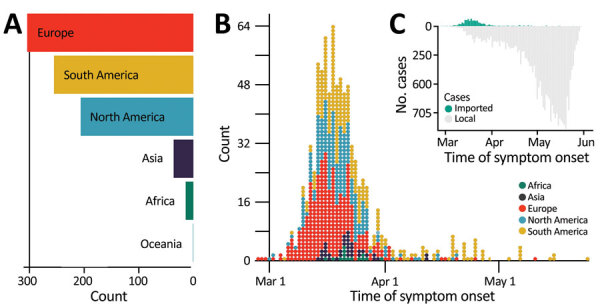
Proportion of imported and local cases early during the COVID-19 pandemic, Colombia. A) Region of origin for the reported imported cases. B) Distribution over time of symptomatic imported and local cases, by region of origin. C) Number of local and imported COVID-19 cases over time. COVID-19, coronavirus disease.

In Colombia, preventive isolation and monitoring for passengers arriving from China, Italy, France, and Spain started on March 10, 2020. A national health emergency was declared on March 12, and tougher measures then started to be set in place, including the closing of borders on March 17, the ban of international flights on March 20, and the ban of domestic flights on March 25. Implementations of lockdowns occurred from March 25 onward, including Resolutions 380 and 385 from the Colombian Ministry of Health and Social Protection (*38*,*39*); Decrees 412 and 457 from the Ministry of the Interior (*40*,*41*); and Decree 439 from the Ministry of Transport (*42*). Despite a massive drop in air traffic, >15,500 residents returned to Colombia through humanitarian flights during April–June (*43*). By June 1, >30,000 cases of COVID-19 had been documented in Colombia and 857 cases (2.8%) had been linked to travel abroad (Figure 1, panel A).

Most (816, 95.2%) imported cases were symptomatic. The prominent geographic sources for symptomatic cases were Spain (245 [28.6%] cases), the United States (203 [23.7%] cases), Ecuador (50 [5.8%] cases); Mexico (49 [5.7%] cases), and Brazil (41 [4.8%] cases). The other 41 imported cases were asymptomatic and were detected through contact tracing. Among asymptomatic imported cases, most (16, 39%) were imported from Spain, the United States (13, 31.7%), Brazil (3, 7.3%), and Mexico (2, 4.9%). Overall, most imported cases were from Spain (30.5%), the United States (25.2%), Mexico (6%), Ecuador (5.8%), and Brazil (5.1%). Most symptomatic imported cases were traced back to countries in Europe and the Americas.

The number of symptomatic imported cases steadily increased and peaked on March 14, when local cases were on the rise, but before border closures and the international air travel ban. Our estimate is based on the average incubation time of COVID-19 (*44*) and symptom onset but is 4.8 days earlier than the actual peak on March 18 (Figure 1, panel B). Initial introductions were predominantly linked to Europe; however, both Europe and the Americas were prominent geographic sources of infections during the onset of the epidemic. The introductions after the peak mainly occurred from countries in South America.

### SARS-CoV-2 Diversity

**Figure 2:**
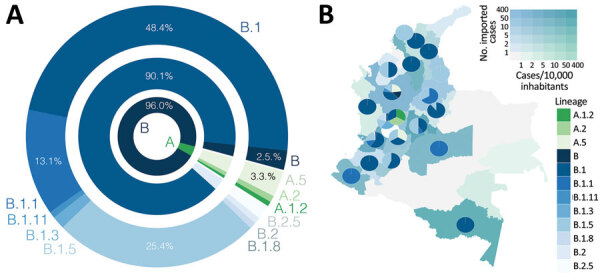
Frequency and distribution of SARS-CoV-2 lineages, Colombia. A) Frequency of A and B lineages and sublineages of SARS-CoV-2 identified. B) Map of distribution of lineages across the country. Departments are colored by the number of imported cases/10,000 inhabitants (inset) and the number of reported introductions. SARS-Co-V-2, severe acute respiratory syndrome coronavirus 2.

To elucidate the dynamics of SARS-CoV-2 spread into Colombia, we combined the 43 whole-genome sequences obtained in our study with sequences from Colombia deposited in GISAID, which provided a set of 122 complete genomes. Sequences from Colombia were classified into 12 sublineages: A.1.2, A.2, A.5, B, B.1, B.1.1, B.1.3, B.1.5, B.1.8, B.1.11, B.2, and B.2.5. The proportion of lineages documented in Colombia seems to reflect founder effects. For example, sublineages B.1, B.1.1, and B.1.5 were found in the early epidemiologically linked transmission chains and consistently were observed most frequently; B.1 was observed in 59 (48.4%) cases, B.1.5 in 31 (25.4%), and B.1.1 in 16 (13.1%) (Figure 2, panel A). From the South America–focused subsampling available from NextStrain, comparable findings were observed for other countries in South America (*45*,*46*), where the most frequently observed lineages were B.1 in 149 (60.8%) cases, B.1.5 in 35 (13.5%) cases, and A.5 in 14 (5.7%) cases.

On average, we identified 1 lineage per department. For instance, the number of documented lineages was highly correlated with the availability of samples (Pearson product-moment correlation coefficient [PPMCC] = 0.72; p<0.001) and uncorrelated with the number of local cases (PPMCC = 0.35; p = 0.049). We noted 5 different lineages in the departments of Valle del Cauca and Antioquia and 3 different lineages in Cundinamarca; these departments have the most populated capitals and we had more samples from them (Figure 2, panel B). We observed a moderate positive correlation between the number of lineages documented in a department and the number of imported cases (PPMCC = 0.51; p = 0.002).

### Molecular Evolution of SARS-CoV-2 in Colombia

We identified 133 single-nucleotide variants (NVs) by using the full genome sequences from Colombia and the reference sequence (GenBank accession no. NC_045512.2). Most NVs (131; 98.5%) fell into the coding region, and 1 NV was identified at each noncoding end. Among NVs in coding sites, 71 (54.2%) led to nonsynonymous substitutions. Most NVs (92/133) were unique to a sequence. Among the shared NVs, 38/41 were associated with a specific lineage (Appendix 1 Tables 3, 4). These observations suggest that the substitutions are not laboratory-specific and most likely the outcome of in situ evolution, shared ancestry, or both (Appendix 2).

In our study, among sequences with complete metadata, 90% (108/120) of sequences from Colombia displayed an amino acid change in region D614G, and the remaining 10% (12 sequences) displayed a change in region D614 (Appendix 1 Table 4). G614 has been associated with higher infectivity (L. Zhang et al., unpub data, https://www.biorxiv.org/content/10.1101/2020.06.12.148726v1) and greater transmissibility with no effects on disease severity outcomes (*46*; E.M. Volz et al., unpub data, https://www.medrxiv.org/content/10.1101/2020.07.31.20166082v2). All G614 sequences also carried mutations that segregate together as described in B. Korber et al. (*47*); we identified the nucleotide substitution C241T at 5¢-UTR; the synonymous substitution C3037T at open reading frame 1ab (ORF1ab), the nonstructural protein 3 encoding-gene; and a change in P4715L aa position in ORF1ab, the RNA-dependent RNA polymerase encoding gene. The presence of these and other mutations can be phenotypically and epidemiologically relevant and warrant further monitoring.

Most patients from Colombia for whom genomic sequences were available were symptomatic (n = 90); 59.6% had cough and fever and the others had >1 symptom; 10 died, 70% of whom had underlying conditions (Appendix 1 Table 1). However, given the limited number of sequences available, we could not reliably investigate any genomic determinant of clinical outcome.

### Evolutionary Relationships between Local and Global SARS-CoV-2 Isolates

**Figure 3:**
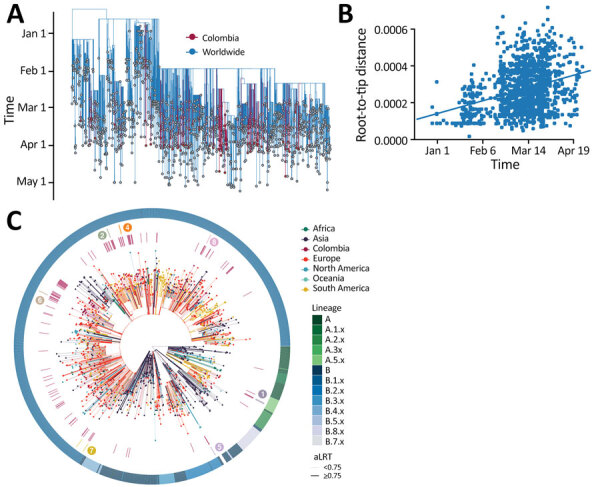
Phylogeny of SARS-CoV-2, Colombia. A) Time-resolved maximum-likelihood tree of 1,578 SARS-CoV-2 sequences. Red indicates 122 sequences from Colombia. B) Root-to-tip distance regression for the sequence data in A. C) Time-resolved maximum-likelihood tree, annotated by region of isolation. The outer ring represents SARS-CoV-2 lineages; the inner red ring highlights the relative position of the sequences from Colombia; the middle ring and the corresponding numbers indicate sequences from epidemiologically linked transmission chains. Branches are colored by the geographic attribution from the migration inference. Highly supported groups are delineated by thicker solid lines. A detailed maximum-likelihood tree is available at https://itol.embl.de/tree/8619015795401231596483440. SARS-CoV-2, severe acute respiratory syndrome coronavirus 2.

The time-stamped phylogeny of 122 isolates from Colombia and 1,462 representative global SARS-CoV-2 isolates showed that the estimated time to the most recent common ancestor for the sampled sequence data is December 7, 2019 (range October 25–December 26, 2019) (Figure 3, panel A). Asia was the inferred ancestral state at the root. Both these observations are in line with the known epidemiology of the pandemic. A root-to-tip regression of genetic distance against sampling time evidenced consistent temporal signal in the sequence data (Figure 3, panel B). The isolates from Colombia were interspersed among the isolates from other countries, suggesting multiple introductions (Figure 3, panels A, C). However, considerable phylogenetic uncertainty appears along the tree and the fine-grained relationships of the isolates from Colombia could not be resolved with confidence (Appendix 2 Figure 1).

**Figure 4:**
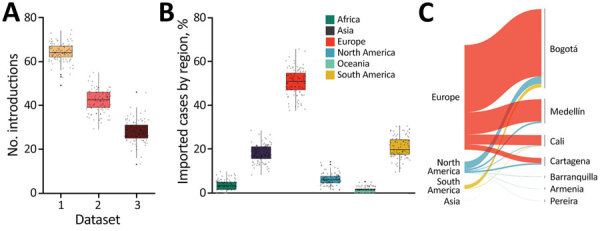
Potential routes of importation for SARS-CoV-2, Colombia. A) The number of transition changes into Colombia following migration inference by using all available sequences per region (dataset 1); retaining several sequences per region, when possible, equal to the number of sequences available for Colombia (dataset 2); and 50 sequences per region and all sequences from Colombia (dataset 3). Box top and bottom lines indicate 25th and 75th percentiles; horizontal lines within boxes indicate means; error bars indicate SDs. B) Geographic source attribution for every transition into Colombia derived from the migration inference using all the available sequences per region. Box top and bottom lines indicate 25th and 75th percentiles; horizontal lines within boxes indicate means; error bars indicate SDs. C) Geographic contribution inferred by using air travel data per country aggregated by region.

Phylogenetic uncertainty and uneven sampling made the quantification of the number of introductions into the country challenging, let alone dating the time of the introductions. The number of state transitions into Colombia heavily relies on the number and nature of the sequences included from other locations (Figure 4, panel A). By using all sequences in the South America–focused subsampling available from NextStrain, we estimated that an average of 64 (interquartile range [IQR] 62–67) introductions into the country have occurred but this estimate gets lower as we reduce the number of samples (sensitivity analyses) from other locations, down to 22 with the most even downsampled dataset. Independent of the dataset, either the complete or the subsampled datasets, and in line with the epidemiologic information, most geographic source attributions are from Europe (Figure 4, panel B; Appendix 2 Figure 2). This observation also aligns with our estimates using travel data (Figure 4, panel C; Appendix 2 Figure 2).

During January–March 2020, a total of 7 cities in Colombia received 1,593,211 international passengers from 14 countries. Bogotá was the most concentrated city for flights, receiving around 77% of passengers; other cities included Medellín with 11%, Cartagena with 6%, and Cali with 4% of passengers. In total, 35% of international passengers started their journeys in the United States, 17% in Mexico, and 12% in Chile. However, we estimate 87% of all imported COVID-19 cases in Colombia came from Europe, 9.5% from North America, and 3.4% from South America. When stratified by country, the primary source of importation was Spain, which had 71.4% of imported cases; the United States had 8.4%, Germany had 8%, and France had 3.4% (Appendix 2 Figure 2). Our data show most (65.2%) COVID-19 cases were among travelers arriving in Bogotá; 20% were among those arriving in Medellín, and 9% among those arriving in Cali. We estimate that the Spain–Bogotá route carried 42% of the total imported cases.

Since the first COVID-19 case was identified in Colombia on February 26, 2020, contact-tracing efforts had been put in place. We obtained multiple sequences from 7 distinct early epidemiologically linked transmission chains (Appendix 1 Table 1) and mapped these data into the phylogeny (Figure 3, panel C). All but 1 set of sequences did not group, but it appeared very close in the tree. These data underscore the potential utility of genomic epidemiology to link persons with incomplete information, such as cases that are disconnected due to intermediate asymptomatic carriers, and complement outbreak transmission investigations.

Our study has some limitations. First, the geographic sources of infection relied on persons self-reporting symptom onset and travel histories, which are subject to inaccuracies. Second, we used air travel data from likely destinations in Colombia, but other locations also might have fueled COVID-19 emergence and dissemination in the country; flight travel data was not available for dates after March 9, 2020. Third, the number of sequences sampled represented a tiny fraction of the documented number of imported cases into Colombia. The sample was selected as a countrywide representation, given limited resources for genome sequencing; thus, the introduced viral diversity also might have been underestimated. Another limitation is the inherent uncertainty stemming from global unsystematic sampling. Therefore, the inferences about the number of introductions and the corresponding geographic sources should be interpreted with caution. We attempted to overcome this by undertaking sensitivity analyses and contrasting the results with the available epidemiologic data and our estimates from travel data. However, more sequence data from Colombia and undersampled countries, together with information of sampling representativeness per country, are needed to account for sampling uncertainty in a more statistically rigorous manner.

## Discussion

We describe the complete genome sequences of SARS-CoV-2 from 43 clinical samples, results of an epidemiologic investigation of imported cases, and the phylogenetic findings of 122 genome sequences from Colombia that characterize the epidemic onset of COVID-19 in the country. Our study provides evidence that several independent COVID-19 introductions occurred in Colombia and documents >12 SARS-CoV-2 lineages. Most of the notified introductions to countries in Latin America occurred from Europe, an observation that was supported by phylogenetic and air travel data (*48*; C. Salazar et al., unpub data, https://www.biorxiv.org/content/10.1101/2020.05.09.086223v1). Although the sequence data do not represent the actual number of epidemiologically linked transmission chains, our phylogenetic findings validated the linkage for epidemiologically linked transmission chains with available sequence data. Our results further underscore the advantages of genome sequencing to complement COVID-19 outbreak investigations and support the need for a more comprehensive country-wide study of the epidemiology and spread of SARS-CoV-2 in Colombia.

Appendix 1 Metadata of patients with diagnosed coronavirus disease, Colombia; GISAID’s nCoV-19 Acknowledgements; nucleotide substitution patterns of the different lineages of SARS-CoV-2 circulating in Colombia; amino acid change patterns of the different lineages of SARS-CoV-2 circulating in Colombia; estimates of evolutionary divergence of SARS-CoV-2 over sequence pairs within and between lineages and sublineages from Colombia.

Appendix 2 Additional information on genomic epidemiology of severe acute respiratory syndrome coronavirus 2, Colombia.
