# Supplementary material for: Genomic Epidemiology of Severe Acute Respiratory Syndrome Coronavirus 2, Colombia
Source: Emerg Infect Dis. 2020 Dec;26(12):2854–62. doi: 10.3201/eid2612.202969 (PMC7706936; doi:10.3201/eid2612.202969)
Supplement: Appendix 2 — Additional information on genomic epidemiology of severe acute respiratory syndrome coronavirus 2, Colombia. [file 20-2969-Techapp-s2.pdf]

Article DOI: https://doi.org/10.3201/eid2612.2969

Genomic Epidemiology of Severe Acute Respiratory Syndrome Coronavirus 2, Colombia

Appendix 2

Potential Routes of COVID-19 Importation in Colombia

For each air travel route (o, d) where o is a country of origin and d a destination, we calculate the proportion 〖E_(o,d)〗 of expected importations along route (o, d) by using the incidence i_O for the country o and the total number 〖P_(o,d)〗_ of passengers on route

$$E_{o,d} = 100 \frac{i_o \, p_{o,d}}{\sum_{(u,t)} i_u \, p_{u,t}}$$

where the sums are taken over all possible routes. Therefore, the proportion of expected E_d importations arriving to Colombia is

$$E_d = 100 \frac{\sum_{u} i_u \, p_{u,d}}{\sum_{(u,t)} i_u \, p_{u,t}}$$

and the proportion of expected importations departing from Colombia is

$$E_o = 100 \frac{\sum_{t} i_o \, p_{o,t}}{\sum_{(u,t)} i_u \, p_{u,t}}$$

Epidemiologic Investigation Per Lineage

Lineage A.1.2 was detected in the city of Manizales from an imported case in a person arriving on March 3 from the United States. Lineage A.2 was identified in the Anserma municipality from a sample collected on March 26, according to the previously published data available at GISAID. Associated traveling and contact-tracing history was not available.

Lineage A.5 was identified in a transmission chain of 3 persons in the city of Medellin without travel history, the first person had symptoms beginning on March 9. These sequences shared a distinctive substitution pattern at the amino acid (aa) G238C at the nucleocapsid and nucleotide, C17470T at ORF1ab helicase, levels (Appendix 1 Tables 2 and 3). A fourth unrelated case from this lineage was identified in the city of Ibague from an imported case in a person arriving on March 17 from an unknown country. Two independent introductions of SARS-CoV-2 lineage A.5 could explain their current epidemiology in Colombia.

We assigned some SARS-CoV-2 sequences to lineage B was assigned because the genetic variability did not enable assignment to a specific sublineage. One patient, Colombia/Cali/79449, arrived in the city of Cali from Spain on March 7. The viral sequence displayed a very similar pattern to lineage B.2, with 2 aa changes, L3606F at open reading frame 1ab (ORF1ab; Nsp6) and G251V at ORF3a (Appendix 1 Table 3). The other 2 patients were identified in the same urban area, Medellín and Sabaneta municipalities belong to the Aburra Valley metropolitan area; the first of the 2 had symptoms beginning on March 13. These results suggest 2 independent introduction events to explain the current presence and distribution of SARS-CoV-2 basal lineage B in Colombia.

Among SARS-CoV-2 sequences analyzed from Colombia, lineage B.1 represented 48%. The genomic analysis of the genetic variability at the aa level revealed 4 substitution patterns (Appendix 1 Table 3), which increased to 5 when analyzed at the nucleotide level (Appendix 1 Table 2). The first pattern was identified in 2 sequences from the city of Neiva belonging to the same transmission chain without travel history. The second substitution pattern was identified mainly in 8 municipalities from the Valle del Cauca department and most sequences were collected from the capital city, Cali. These sequences displayed a distinctive aa change in T265I at ORF1ab (Nsp2) (Appendix 1 Table 3). Eight patients reported international travel history from Spain and the United States and the earliest arrived on March 6, 2020. The third substitution

pattern mainly was distributed in the Caribbean region, in the cities of Ciénaga, Monteria, Santa Marta, and Valledupar, and municipalities from the center of the country near the capital, Bogotá. The first case belonging to this pattern corresponded to an imported case in a person entering the country from Spain on March 1, early during the pandemic in Colombia and previous to the domestic and international flights restriction. The fourth substitution pattern was identified in several municipalities without geographic proximity, including Medellín, Cartagena, Ibague, Leticia, and Togui (this case was reported in a person with recent travel to a national tourist region), from confirmed cases without international travel history from the available data. The first case among this group of sequences was identified in Cartagena on March 10, 2020. Finally, the fifth substitution pattern was identified in 3 sequences from samples collected in the cities Neiva and Envigado, which are in different regions of the country. The first patient reported symptoms that began on March 18, 2 days after the person entered Colombia from Panama. These sequences displayed a distinctive aa change in I71V at ORF1ab (leader). Patterns 2–5 shared the aa change Q57H at ORF3a; patterns 3–5 shared the synonymous substitution C18877T, 3′-to-5′ exonuclease; and patterns 4 and 5 shared the synonymous substitution C10509T, Nsp5A (Appendix 1 Tables 2 and 3).

Lineage B.1.1 was not directly assigned by PANGOLIN (https://github.com/cov-lineages/pangolin), however it was manually defined for those sequences classified as B.1 that possessed the substitutions G28881A, G28882A, G28883C at the nucleocapsid gene leading to the aa changes R203K and G204R (Appendix 1 Table 3; https://github.com/hCoV-2019/lineages) and corroborated through GISAID assignment. This lineage was represented by 16 sequences, including the first confirmed case of COVID-19 in a person entering the country from Italy on February 26 and displays 5 nucleotide substitutions common to pairs of sequences, 4 of which also displayed geographic correspondence; thus, representing previously identified or unidentified local transmission chains. Two sequences obtained from patients from Villavicencio city without traveling history belonged to a previously characterized transmission chain and displayed 2 distinctive aa changes, S2488F at Orf1ab (Nsp3) and T14A at ORF7a (Appendix 1 Table 3).

Lineage B.1.3 was identified in the city of Pereira and the municipality of Dosquebradas, which belong to the same metropolitan area. The patients had no traveling history and the first one had symptoms beginning on April 2. Despite the lack of available information about the

epidemiologic relationship between the 2 patients, the fact their sequences shared the same nucleotide substitution pattern, geographic distribution, and temporality suggest that they belong to the same transmission cluster.

Lineage B.1.5 was the second most frequently noted sequences, accounting for the 26% of the analyzed sequences from Colombia. Any aa change is distinctive of this lineage, but all the sequences shared the exclusive synonymous substitution of A20268G at ORF1ab (endoRNase). The analysis of the genetic variability at the amino acid level revealed 3 substitution patterns (Appendix 1 Table 3), which increased to 5 when analyzed at the nucleotide level (Appendix 1 Table 2). The first substitution pattern was represented by sequences from the municipalities of Barranquilla, Bello, Bogotá, Cali, Cucuta, Medellín, Neiva, Pacho, Palmira, and Tierralta, located in 7 different departments. Despite the lack of geographic clustering, 12/18 patients belonging to this group reported entering the country from Spain, France, or Italy. Of note, 5 patients entered on March 9, 2020 and 3 entered on March 12, 2020. The second pattern was defined by the presence of the synonymous substitution C23443T in the Spike protein gene. According to the available information from 2 of the patients, no travel history was reported. Pattern 3 sequences shared the G29734C substitution at the 3′UTR and was distributed in Pereira, Cali, and Yumbo, belonging to the interconnected departments Valle del Cauca and Risaralda. The first patient also reported entering the country from Spain on March 9, 2020 and having symptoms on the same day. The fourth pattern was identified in the city of Cali from 2 patients belonging to the same transmission chain who shared the aa change R191C at the Nucleocapsid protein and the synonymous substitution C1327T at Orf1ab (Nsp2). The fifth substitution pattern was defined by two distinctive aa changes at Orf1ab, at A3610V in the Nsp6 and at G5063S in RdRp genes. This pattern was identified in Cali city and Buga municipality, both in the department of Valle del Cauca, the first patient reported travel history from Spain on March 13, 2020.

Lineage B.1.8 was identified in the city of Pereira from a single sequence available at GISAID obtained from a patient arriving from Spain on March 15. Lineage B.1.11 was identified in Itagui (Aburra Valley) from a patient who reported symptoms starting on March 23. Lineage B.2 was identified in the city of Cali from a single sequence obtained from a patient without travel history who reported symptoms beginning on March 29. Finally, lineage B.2.5 was

represented by a previously referred transmission chain with the first patient arriving in Armenia on March 10, from Italy.

Evolutionary Divergence Within and Between Lineages

The estimate of average evolutionary divergence between sequence pairs of lineages A and B was 0.000401 base substitutions per site but estimates for within each lineage sequence pairs were 0.000169 for lineage A and 0.000222 for lineage B (Appendix 1 Table 4). For a better resolution in the estimation of evolutionary divergence within and between groups, sequences were grouped according to different sublineage levels. The analysis at sublineage level 1 enable sequences to be grouped into A.x and B.x and at sublineage level 2, the grouped as belonging to A.x.x and B.x.x. These comparisons at sublineage level 1 enabled us to identify a higher estimated evolutionary divergence within sublineages B.1 and B.2. The estimates of genetic distance between sublineages was higher for comparisons of A.2 with the other sublineages. Of note, the average divergence between sublineages assigned to the same lineage, such as (A.1 vs. A.2 or A.2 vs. A.5, was higher than other comparisons between sublineages assigned to different lineages, such as A.1 vs. B.1, A.1 vs. B Basal, or A.2 vs. B Basal. The higher resolution comparisons at sublineage level 2 enabled us to identify the higher within-sublineage divergence for B.1.1 and B Basal. The estimates of evolutionary divergence between sublineages showed higher values when A.2 Basal was compared with any sublineage from the B lineage and also when B.2.5 was compared with any other sublineage. The average divergence between sublineages assigned to the same lineage, including A.1.2 vs. A.2 Basal, A.2 Basal vs. A.5 Basal, B.1.1 vs. B.2.5, and B.1.3 vs. B.2.5, was higher than other comparisons between sublineages assigned to different lineages, such as A.1.2 vs. B.1.5, B.1.8, B.1.11, B.1 Basal, B.2 Basal, or B Basal (Appendix 1 Table 4). These results can be affected by the low sequence number for some lineages, but the estimates of the evolutionary distance between groups are in agreement with the lineage assignment and with the substitution patterns observed to be unique to each lineage (Appendix 1 Tables 3, 4).

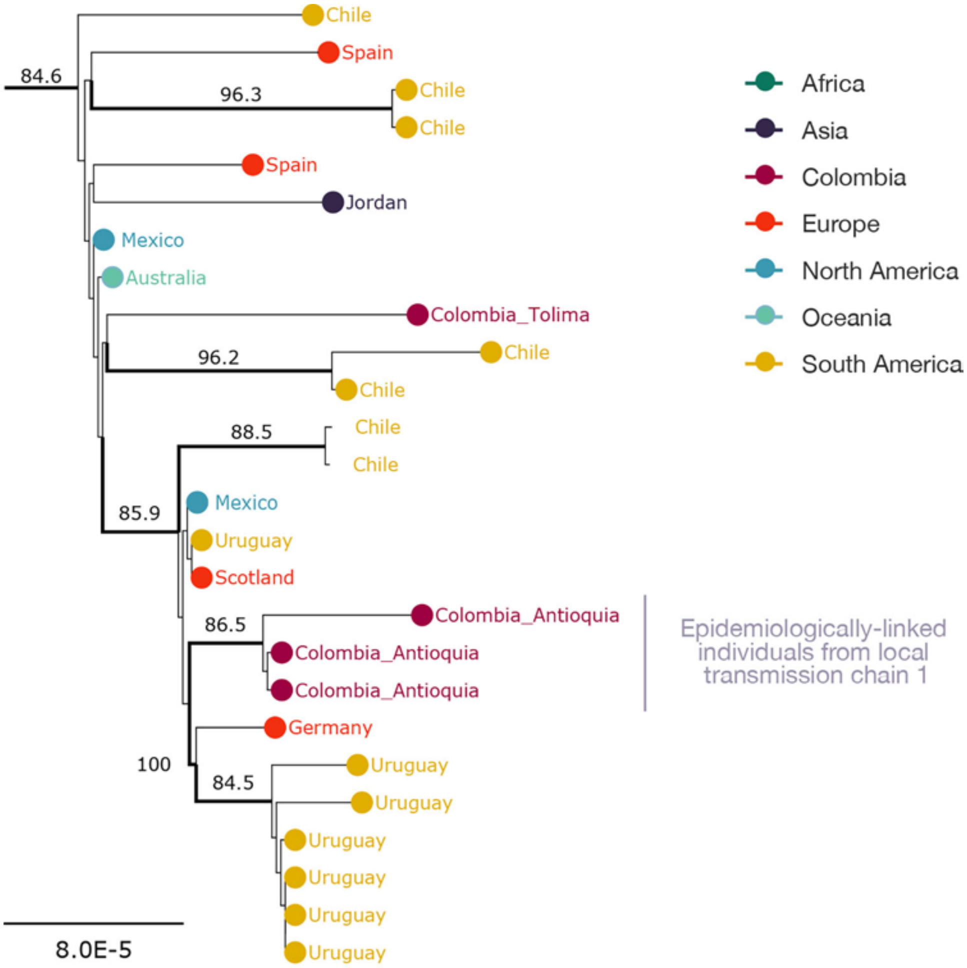

Appendix 2 Figure 1. Example of a SARS-CoV-2 clade with sequences from a local transmission chain from Colombia. Tips are labeled by country (and department in the case of isolates from Colombia) and colored by region. Of note, fine-grained relationships could not always be resolved with confidence. Branch supports with aLRT <0.75 are not shown. Scale bars indicate number of nucleotide substitutions per site. aLRT, approximate likelihood ratio test; SARS-CoV-2, severe acute respiratory syndrome coronavirus 2.

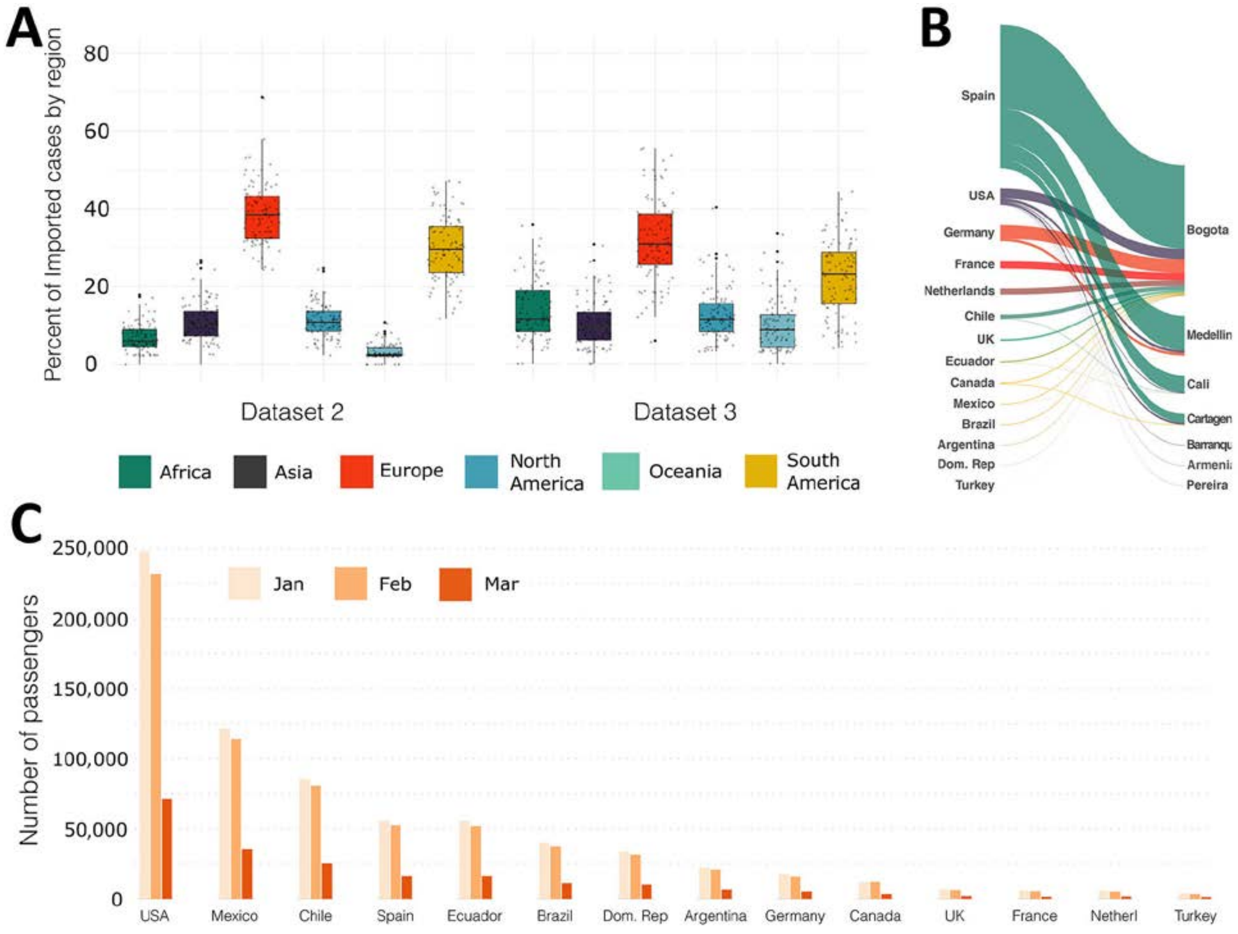

Appendix 2 Figure 2. Potential countries of introductions of SARS-CoV-2 to Colombia. A) Geographic source attribution for every transition into Colombia derived from the migration inference retaining several sequences per region, when possible, (dataset 2) equal to the number of sequences available for Colombia and retaining 50 sequences per region and all sequences from Colombia (dataset 3). Error bars show SDs; horizontal lines indicate means. B) Number of passengers arriving in Colombia from different countries. C) Geographic contribution inferred by using air travel data per country.
